# Transcriptional response to combination antiretroviral therapy predicts side effects and novel targets

**DOI:** 10.3389/fphar.2025.1743543

**Published:** 2026-01-21

**Authors:** Alexander Lachmann, Letizia Amadori, Paola Nicoletti, Heidi M. Crane, Chiara Giannarelli, Avi Ma’ayan, Inga Peter

**Affiliations:** 1 Department of Pharmacological Sciences, Icahn School of Medicine at Mount Sinai, New York, NY, United States; 2 Department of Artificial Intelligence and Human Health, Icahn School of Medicine at Mount Sinai, New York, NY, United States; 3 Mount Sinai Center for Bioinformatics, Icahn School of Medicine at Mount Sinai, New York, NY, United States; 4 Leon H. Charney Division of Cardiology, Department of Medicine, New York University Cardiovascular Research Center, New York University Grossman School of Medicine, New York University Langone Health, New York, NY, United States; 5 Department of Genetics and Genomic Sciences, Icahn School of Medicine at Mount Sinai, New York, NY, United States; 6 Department of Medicine, University of Washington, Seattle, WA, United States

**Keywords:** adverse drug effect, antiretroviral therapies, *in vitro* cell line treatment, *in silico* prediction, LINCS L1000, transcriptomic profiling

## Abstract

**Background:**

Antiretroviral therapy (ART) has revolutionized the clinical management of people with human immunodeficiency virus (HIV), transforming HIV infection into a chronic condition. Yet, the mechanisms of action and off-target effects of modern combination ART regimens versus individual ART medications are not fully understood.

**Methods:**

Using the L1000 assay, we profiled transcriptional responses to 11 single ART drugs and 6 ART combination regimens across three human cell lines, HepPG2 (liver), HK2 (kidney), and THP-1 (monocyte). Differentially expressed genes were analyzed against host-HIV protein-protein interactions (PPIs) and genes implicated in ART-associated side effects.

**Results:**

Across all cell types, ART combination regimens induced distinct transcriptional profiles compared with their component drugs. Combinations more strongly perturbed genes encoding proteins involved in HIV–host PPIs, consistent with their enhanced antiviral efficacy. Transcriptional responses also recapitulated known ART-induced adverse effects related to dyslipidemia, altered body composition, and renal impairment. Combination regimens were less coupled to these gene signatures, suggesting mechanisms that may underlie their improved safety profiles. Several genes and pathways were consistently modulated across treatments: *ACTG1*, or actin gamma 1 — a gene that encodes gamma actin, a protein crucial for the localization of the HIV reverse transcription complex, was downregulated in four combination regimens, while *ORM2*, Orosomucoid 2, upregulation emerged as a common response to individual drugs. *ACTG1* was previously found to be downregulated in ART naïve people living with HIV who naturally control HIV replication, suggesting its role as a candidate host mediator. To facilitate data exploration, we developed *ARTexpress*, an interactive portal enabling visualization of gene expression changes before and after ART exposure across all three cell lines.

**Conclusion:**

ART regimens affected transcriptional signatures of genes involved in HIV-host PPIs and were less tied to common ART-related side effects. Our findings support the use of high-throughput transcriptomics to detect specific mechanisms of ART on- and off-target effects to help prioritize new drug targets and compounds in future development and optimization of safer and more efficient ART.

## Introduction

**FIGURE 1:**
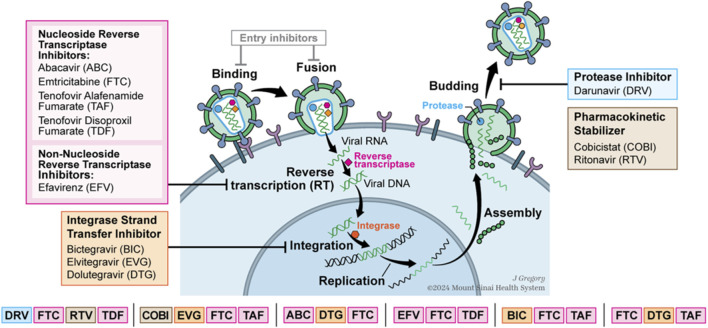
HIV lifecycle and drug actions are shown relative to lifecycle. Drugs are categorized into reverse transcriptase inhibitors, integrase strand transfer inhibitors, protease inhibitors, and pharmacokinetic stabilizer.

Antiretroviral therapy (ART) has transformed human immunodeficiency virus (HIV) infection into a manageable chronic disease, greatly reducing progression to acquired immunodeficiency syndrome (AIDS) and related morbidity and mortality in people with HIV (PWH) (Deeks et al., 2013). Several ART drug classes target distinct stages in the HIV life cycle (Figure 1). Currently, the most commonly used ART medications are 2nd generation integrase strand transfer inhibitors (INSTI) that block HIV integrase from inserting viral DNA into the host DNA and nucleoside reverse transcriptase inhibitors (NRTI) blocking the viral enzyme reverse transcriptase to stop HIV replication (Ma et al., 2022; Azzman et al., 2024). Other ART classes that have been commonly used include protease inhibitors (PI), which prevent the cleavage of viral protein precursors into functional proteins that are essential for viral replication, and nonnucleoside reverse transcriptase inhibitors (NNRTI) that also block viral enzyme reverse transcriptase although through a different mechanism than NRTIs. Additional ART classes include fusion, entry, or post-attachment inhibitors that block HIV from entering host immune cells by binding to cell surface receptors.

Despite a positive impact on clinical progression and death, and efficacy in suppressing the viral load at least for a short time, early monotherapy regimens such as using zidovudine had high pill burden, lack of prolonged viral suppression, and treatment limiting toxicities and side effects and frequently resulted in the emergence of multiple drug resistance mutations (Tseng et al., 2015). Combination ART regimens targeting difference stages of the HIV life cycle have revolutionized HIV management, reducing acquired drug resistance, extending viral suppression, and improving tolerability (HIV-CAUSAL Collaboration, 2010). As a result, the standard practice evolved into combining three ART medications from at least 2 different classes that suppress HIV viral load, often with a pharmacokinetic enhancer that reduces the rate-controlling steps in the metabolism of the core drug or inhibits its inactivation (Tseng et al., 2015). Clinical trials and observational studies indicate that newer ART regimens offer improved safety profiles compared to older ART regimens. However, while pharmacokinetic enhancers allow for reduced dosing of individual ART drugs, questions remain regarding the extent these combinations reduce toxicity by offsetting off-target effects (Panel on Clinical Practices for Treatment of HIV Infection, 2001). Moreover, the clinical data on the side effects of different ART regimens is largely observational or from trials with specific populations and limited size, complicating efforts to identify molecular pathways responsible for adverse effects of either single-drug or combinatorial ART regimens, given the potential for long-term toxicities.

Connectivity Mapping is a promising approach for elucidating the molecular mechanisms of both single drugs and their combinations by assessing their effects on the transcriptome of tissue-relevant human cell lines at high-throughput (Keenan et al., 2019). As part of the NIH Common Fund Library of Integrated Network-based Cellular Signatures (LINCS) program, the L1000 assay evaluated the response of 278 human cells to ∼33,000 chemical perturbations and ∼8,000 single gene knockouts, resulting in ∼4 million gene expression profiles (Keenan et al., 2018; Subramanian et al., 2017). The L1000 assay measures the expression of 978 landmark genes, selected for their representativeness of the entire transcriptome, while inferring the expression of over 11,000 additional genes computationally. In our prior work, we leveraged publicly available LINCS L1000 profiles of 15 single ART compounds, revealing that the RNA processing machinery, identified in an atherosclerotic arterial wall, was consistently enriched among the differentially expressed gene signatures induced by several PIs (Frades et al., 2019). When we treated cholesteryl ester-loaded THP-1 cells, an *in-vitro* atherosclerosis model, with the PIs ritonavir, nelfinavir, or saquinavir, we observed a doubling of cholesteryl ester accumulation. In contrast, RNA silencing of the subnetwork’s top key driver, polyglutamine binding protein 1 (*PQBP1*), reduced cholesteryl ester accumulation after treatment with any of these drugs. These findings suggest a mechanism that may underlie the increased risk for coronary artery disease in PWH treated with PIs. This study highlighted the utility of the L1000 assay for elucidating the cellular-level molecular mechanisms of ART-associated side effects.

However, despite the necessity for lifelong treatment with ART in PWH, our understanding of how newer combinations of three or four medications cause adverse events remains limited. Understanding the mechanisms of action and off-target effects of modern ART combinations is essential for optimizing therapy and minimizing long-term risk of adverse events (Duwal and von Kleist, 2016). Therefore, we analyzed the response of human liver, kidney, and monocyte cell lines to common single drug ART medications and clinically relevant combination regimens using the L1000 assay to identify transcriptional signatures induced by ART.

Our aim is to identify cellular pathways that may promote off-target drug effects linked to adverse events induced by current and future ART regimens, thereby leading to improved personalized treatment options for PWH.

## Materials and methods

### Cell line experimentation

**FIGURE 2:**
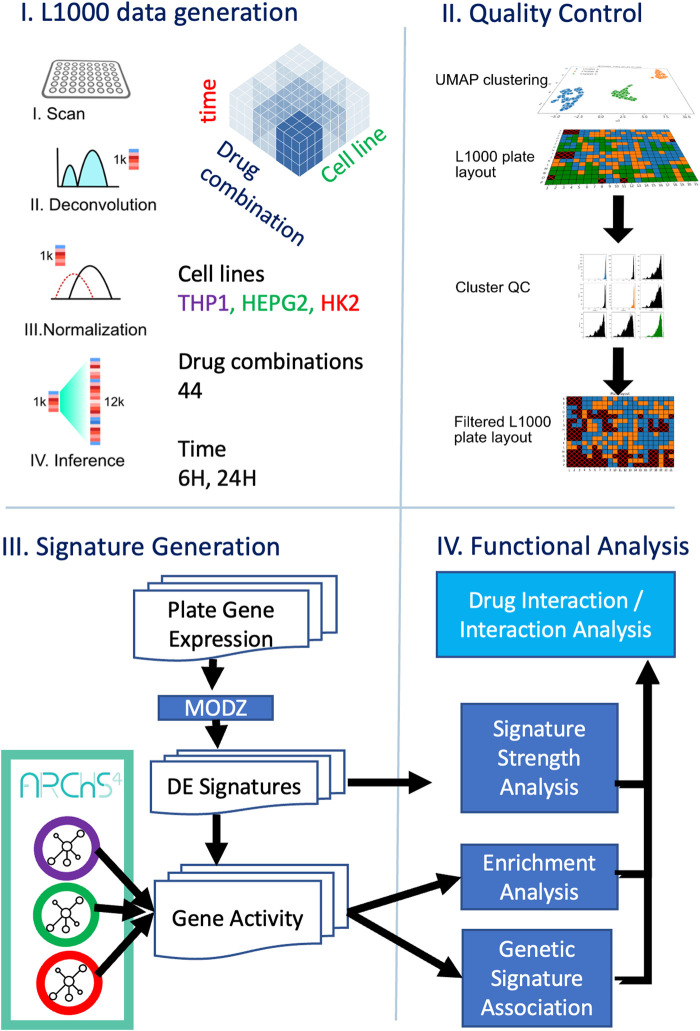
Workflow of antiretroviral therapy signature analysis of L1000 gene expression data.

We selected 11 ART medications commonly used in combination regimens, of which 10 are currently prescribed and one is no longer commonly used in the U.S. but at one time was the core drug for the single most common ART regimen, to experimentally evaluate their effect on the three cell lines (Figure 2). The dose selection was based on the conversion from human equivalent dose used in clinical settings to dosages applicable to *in vitro* settings, using the body surface area normalization method (Reagan-Shaw et al., 2008). The 11 medications were applied to the cell lines in clinically relevant conditions, with drug dosages titrated and tested for viability (Cell Titer Fluor, Promega, cat. no. G6081), cytotoxicity (LDH-Glo Cytotoxicity Assay, Promega, cat. no. J2381) and apoptosis/necrosis (RealTime-Glo Annexin V Apoptosis and Necrosis Assay, Promega, cat. no. JA1012), following manufacturer’s instructions. The final concentration was selected based on both the *in vitro* testing results and by referring to dosages previously used in the same cell line settings (see Supplementary Methods). The selection of the three cell lines was based on their relevancy to widely observed adverse events. The human monocytic cell line THP-1 serves as an *in vitro* model for foam cell formation, which is involved in atherosclerosis at all stages of development; Human Kidney-2 (HK-2) is a model for kidney disease; and hepatoblastoma-G2 (HepG2) is a model for liver disease. The human cell lines used in this study were obtained from the American Type Culture Collection (ATCC) and cultured in 384-well plates according ATCC’s instructions. Briefly, THP-1 cells (10,000 cells/well; ATCC, cat. no. TIB-202) were cultured in RPMI-40 medium (Gibco, cat. no. 11875-093) supplemented with 10% fetal bovine serum (FBSGibco, cat. no. 10438026) and 1% penicillin/streptomycin (P/S, Corning, cat. no. 30-002) at 37 °C, 5% CO_2_. Macrophage differentiation was induced *in vitro* with phorbol-12-myristate-13-acetate (PMA, 50 ng/mL; Sigma) for 72 h at 37 °C in 5% CO_2_, as described (Tsuchiya et al., 1982; Ibanez et al., 2012; Talukdar et al., 2016). HK-2 cells (5,000 cells/well; ATCC, cat. no. CRL-2190) were cultured in Keratinocyte Serum Free Medium (K-SFM), Kit (Gibco, cat. no. 17005-042) at 37 °C, 5% CO_2_. HepG2 cells (5,000 cells/well; ATCC, HB-8065) were cultured in Eagle’s Minimum Essential Medium (EMEM; ATCC, cat. no. 30-2003), supplemented with 10% FBS and 1% P/S at 37 °C, 5% CO_2_. After 24 h of culture for HepG2 and HK-2 cells, and 72 h for THP-1 macrophages, the cells were placed in their respective culture media supplemented with 2% FBS for 16 h. Subsequently, they were treated for 6 or 24 h with either vehicle (DMSO) or with ART medications at a concentration of 0.1, or 0.5 µM (Supplementary Table S1). For all treatments, three biological replicates were used for each cell line per condition. After treatment, cells were lysed using the TCL Buffer (Qiagen, cat. no. 1031576) and incubated for 30 min at 37 °C. The lysates were then stored at −80 °C. This study does not involve human subjects as defined by federal guidelines, and therefore did not require Institutional Review Board (IRB) review.

### L1000 gene expression data generation

Gene expression was quantified using the L1000 assay, a high-throughput profiling method previously described (Subramanian et al., 2017) (Figure 2). This approach directly measures the expression of 978 landmark genes. The resulting dataset was then normalized relative to the expression of 80 invariant landmark control genes. In this assay, the expression of additional genes is inferred via a linear model trained on thousands of Affymetrix gene expression datasets from the gene expression omnibus (GEO). In this study, we utilized the L1000 data with the 12,328 measured and inferred mRNAs.

### Quality assurance of L1000 gene expression profiles

Initial data quality assessment was performed using the L1000 preprocessing pipeline. To ensure internal consistency, touchstone perturbations were included on the plates, utilizing drugs with well-characterized, robust, and highly reproducible effects, such as Vorinostat (Supplementary Methods; Supplementary Figures S1–S3). To validate the quality of L1000 gene expression profiles with the use of external data, we conducted a quality control (QC) and filtering process by comparing them to publicly available ARCHS4 (Lachmann et al., 2018) reference profiles for the same cell lines HK2, HepG2, and THP-1. This step aimed to identify and exclude L1000 samples that diverged from the expected gene expression patterns of these cell lines, preserving only reliable profiles for further analysis. Using the archs4py Python package, we retrieved ARCHS4 gene expression profiles via metadata searches with the terms “HK2,” “HepG2,” and “THP-1“ to match samples to each cell line. For every L1000 profile, we computed its correlation with the corresponding ARCHS4 profiles by focusing on the 11,888 overlapping genes and aligning plates to their respective cell lines. We then calculated the average correlation of each L1000 profile with all matching ARCHS4 samples. Next, a Gaussian mixture model (GMM) with two components (k = 2) was applied to separate the profiles into clusters of higher and lower average correlation to the ARCHS4 references. We discarded all samples in the low-correlation cluster, retaining only those with strong alignment to the ARCHS4 profiles (Supplementary Methods; Supplementary Figure S4). This process ensures that only high-quality L1000 data, consistent with established expression patterns, are used in subsequent analyses (Supplementary Figure S5).

### L1000 differential signature generation

To generate replicate-consensus signatures for drug perturbations, we first computed the modulated z-score (MODZ) for each plate after removing gene expression profiles that failed to meet previously established QC standards following the protocol used in the original L1000 publication (Keenan et al., 2018; Subramanian et al., 2017). Each drug combination was tested in triplicate. We began by determining the z-score for each drug perturbation on the plate. Next, we constructed a pairwise Spearman correlation (Wissler, 1905) matrix among all replicates, setting the diagonal self-correlations to NaN to exclude them. Next, we determined the average correlation of each replicate with the others. For the final consensus signature, we summed the z-score signatures, weighting each by its average correlation. Replicates with a negative average correlation were assigned a weight of zero, ensuring that only those with positive correlations contributed to the consensus signature. This method prioritizes reliable replicates in forming the consensus profile.

### Tissue-specific gene activity network analysis

For each of the three gene expression matrices extracted from the ARCHS4 database (THP-1, HepG2, and HK-2), we first filtered genes based on expression levels, requiring a gene to have a count of at least 20 in 10% or more of the samples. This step eliminates genes with low expression and pseudogenes from subsequent analysis. After filtering, the resulting datasets included 16,395 genes across 3,439 samples for HepG2, 21,303 genes across 6,431 samples for HK-2, and 17,619 genes across 2,606 samples for THP-1. We then normalized the gene count data for library size using the Trimmed Mean of M-values (TMM) method (Baguio, 2008). Following normalization, we computed pairwise Pearson correlations for all gene pairs. To construct the gene neighborhood network, we connected genes if they ranked among the top 100 correlated genes for each other. Finally, we calculated the normalized enrichment score (NES) for each MODZ gene expression signature using the gene neighborhood network as the gene set library with blitzGSEA (Lachmann et al., 2022).

### GWAS gene set enrichment analysis and identification of key genes

Genome-wide association study (GWAS) gene sets were obtained from the GWAS Catalog (Sollis et al., 2023) (2019) library via the Enrichr database (Kuleshov et al., 2016). Prior to performing enrichment analysis, we excluded ribosomal genes from the gene expression profiles. These genes exhibit strong correlations with one another, which can lead to their overrepresentation in enrichment results. This step is necessary because enrichment analyses typically assume statistical independence among genes, an assumption that ribosomal genes violate due to their high intercorrelations. In this study, we sought to determine whether gene sets associated with specific biological themes exhibited coordinated up- or downregulation of genes sets associated with known side effects of ART therapy. To this end, we curated groups of gene sets corresponding to common adverse events associated with ART (Supplementary Table S2). To investigate the collective behavior of gene sets, we first performed gene set enrichment analysis (GSEA) using the blitzGSEA method (Lachmann et al., 2022) on a total of 996 GWAS-derived gene sets. This initial step generated a ranking for each gene set, indicating the significance of up- or downregulation of its constituent genes within drug response signatures. Next, we organized these gene sets into biologically related categories, specifically focusing on low-density lipoprotein cholesterol (LDL) levels, body fat composition, and glomerular filtration rate; each category comprised multiple gene sets. To assess whether these thematic groups exhibited coordinated regulation, we applied a secondary enrichment analysis using blitzGSEA on the rankings of the gene sets within each category. This approach determined whether the gene sets within a group were significantly enriched, indicating that the genes in these sets were consistently up- or downregulated in the context of the drug signatures, which indicates a coordinated regulation. Furthermore, to pinpoint potential driver genes underlying these biological effects, we examined the genes most frequently appearing in the leading edges of the enriched gene sets, as identified by the blitzGSEA analysis. This dual strategy allowed us to assess thematic gene set regulation but also identify key genes that contribute to the observed phenotype.

### Developing the ARTexpress signature portal

The ARTexpress Signature Portal was developed in Python 3 using a FastAPI backend. The backend serves signature information based on gene-centric or signature-centric queries. The gene search function lists the gene activity of a gene of interest for all drug perturbations and cell lines. The signature-centric search lets a user specify the cell line, time point, and drug perturbation and returns the corresponding gene activity profile. ARTexpress supports enrichment analysis using single gene sets or paired up/down gene sets. Enrichment analysis of drug signatures is computed using blitzGSEA (Lachmann et al., 2022). For paired gene sets, enrichment is calculated separately for the up and down gene sets, and NES for both gene sets are multiplied to derive a composite enrichment score. The absolute composite enrichment score is reported. Based on directionality, ARTexpress labels drug signatures as either mimickers, reversers, or ambiguous. A signature is a mimicker of a paired gene set query if the “up genes” are upregulated in the signature and the “down genes” are downregulated. A signature is labeled as a reverser when the “up genes” are downregulated, and the “down genes” are upregulated. Otherwise, a signature is labeled as ambiguous; for example, if both the “up genes” and “down genes” are upregulated. For a single gene set enrichment, a signature is marked as a mimicker if the genes in the gene set are upregulated, and as a reverser when the genes are downregulated. Modulated z-score signatures, gene activity profiles, and tissue-specific gene networks are available for download (Supplementary Methods). ARTexpress is openly accessible from: https://maayanlab.cloud/artexpress. The source code of ARTexpress is available at: https://github.com/maayanlab/artsexpress.

## Results

### Signature characterization

A Uniform Manifold Approximation and Projection (Becht et al., 2018) (UMAP) plot of L1000 drug treatment cell line-specific activity profiles for THP-1, HepG2, and HK-2 cell lines at 6-h and 24-h time points showed that drug signatures were clustered by cell type, with no significant differences observed between the two time points (Supplementary Figure S6A). However, the availability of high-quality drug perturbation signatures was not uniform across cell lines and time points, resulting in missing drug perturbations due to removal during the QC step (Supplementary Figure S6B). For example, after filtering gene expression profiles as described in the *Methods*, certain drug effects, like those with Elvitegravir (EVG), only passed QC in THP-1 cell lines at the 24-h mark, showing low similarity to reference cell line profiles and being reported in one specific condition only. Signature strength analysis revealed that ART drug perturbations elicited less pronounced transcriptional responses compared to the touchstone perturbations, leading to reduced reproducibility. On average, touchstone perturbations exhibited significantly higher replicate correlation (Supplementary Figures S1–S3, S5). Some plates, such as those using HepG2 at 24-h, showed low concordance with other HepG2 cell line data collected from ARCHS4, resulting in nearly half the samples being removed in the QC step. In contrast, samples on other plates, such as HK-2 at 6-h, mostly passed QC (Supplementary Figure S4).

### Identification of target genes associated with ART-associated cell response

**FIGURE 3:**
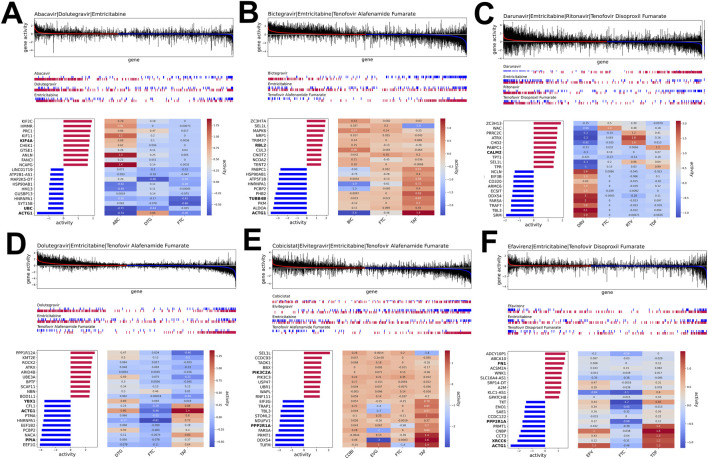
Gene activity profiles for HIV drug combinations. Each subplot displays, from top to bottom: (top) boxplots of gene activities across replicates for all genes ranked by median activity; (middle) indicator bars for the top 250 up-regulated (red) and bottom 250 down-regulated (blue) genes from each individual drug component, aligned to the combination’s gene ranking; (bottom left) bar plot of the top 10 up- and bottom 10 down-regulated genes in the drug combination; and (bottom right) heatmap of their activities under each component drug. **(A)** Abacavir|Dolutegravir|Emtricitabine; **(B)** Bictegravir|Emtricitabine|Tenofovir Alafenamide Fumarate; **(C)** Darunavir|Emtricitabine|Ritonavir|Tenofovir Disoproxil Fumarate; **(D)** Dolutegravir|Emtricitabine|Tenofovir Alafenamide Fumarate; **(E)** Cobicistat|Elvitegravir|Emtricitabine|Tenofovir Alafenamide Fumarate; **(F)** Efavirenz|Emtricitabine|Tenofovir Disoproxil Fumarate.

To investigate whether the transcriptional signatures of single drugs are preserved in the combination ART signatures (Figure 3), we analyzed the response of ART combinations compared to the single drugs within those combinations. We found some of the ART combinations that induced responses similar to those observed for single drugs included in the regimen. For example, the transcriptional response induced by Abacavir (ABC) closely resembled the response by ABC/Dolutegravir/Emtricitabine (ABC/DTG/FTC; Figure 3A), indicating that the effect of ABC dominates that of DTG and FTC in the combination. Similarly, the perturbation signature of Bictegravir/FTC/Tenofovir Alafenamide Fumarate (BIC/FTC/TAF) resembled that of BIC but inversely related to that induced by TAF (Figure 3B). Moreover, the transcriptional signature induced by the combination Darunavir/FTC/Ritonavir/Tenofovir Disoproxil Fumarate (DRV/FTC/RTV/TDF) aligned with the RTV signature while being opposite of the DRV signature (Figure 3C). Additionally, the signature induced by DTG/FTC/TAF was opposite to that of TAF (Figure 3D). In other instances, ART combination signatures were distinct from the individual perturbations within the regimen. For instance, Cobicistat/EVG/FTC/TAF (COBI/EVG/FTC/TAF) and Efavirez/FTC/TDF (EFV/FTC/TDF; Figures 3E,F) displayed transcriptional patterns that differed from those induced by the single drugs, suggesting that the combination ART regimens induce unique gene expression profiles.

Focusing on specific genes targeted by combination ART regimens across different cells, we found that *ACTG1*, or actin gamma 1 — a gene that encodes gamma actin, a protein crucial for the localization of the HIV-1 reverse transcription complex—was consistently downregulated by the four combination ART regimens, ABC/DTG/FTC (Figure 3A), BIC/FTC/TAF (Figure 3B), DTG|FTC|TAF (Figure 3C), and EFV/FTC/TDF (Figure 3F). Additionally, the host-HIV-associated gene, *PPP2R1A*—which encodes the alpha (α) subunit of Protein Phosphatase 2A (PP2A) — was downregulated with COBI/EVG/FTC/TAF (Figure 3D) and DTG/FTC/TAF (Figure 3C). Among the ten most differentially expressed genes in the ART regimens (Figure 3), we identified genes associated with metabolic and cellular processes components, including genes involved in RNA methylation (*PRMT1*), transcription (*DDX54, CNBP*), and glycolysis (*ENO1, ALDOA, and PKM*). Despite these findings, most of the top target genes were specific to treatment regimen. Notably, *EEF1G* and *EEF1B2* exhibited ∼4-fold downregulation in response to DTG/FTC/TAF regimen (Figure 3D). This significant downregulation was unique to the combination signature and was not observed when DTG, FTC, and TAF were used individually.

**FIGURE 4:**
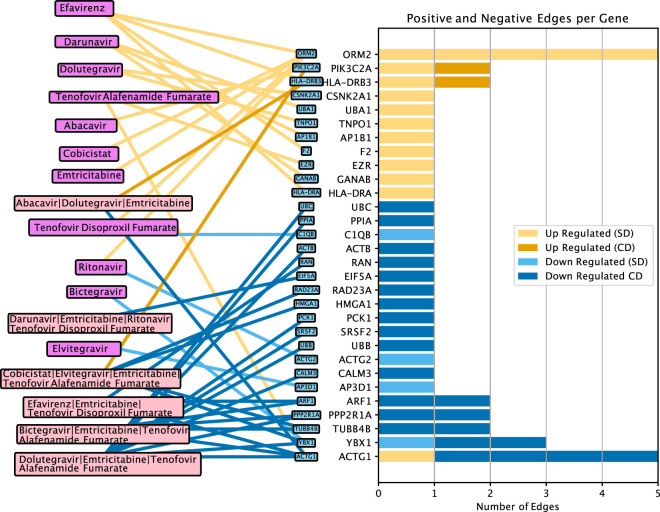
Gene-antiretroviral therapy (ART) interaction network depicting the top differentially expressed genes following various ART treatments across the three tissues. SD, single drug regimen. CD, combination drug regimen.

Next, we investigated whether genes exhibiting the greatest transcriptional response across combination ART regimens influenced cellular metabolic pathways relevant to viral replication and persistence. By examining genes encoding known host-HIV interacting proteins (Chatr-aryamontri et al., 2009) within the ART transcriptional signatures, we discovered that the gene expression signatures of ART combinations, averaged across the cell types, were significantly enriched for genes encoding human proteins known to interact with HIV viral proteins (Figure 4; Supplementary Table S3). This enrichment was more pronounced for combination ART regimens compared to the individual drugs that comprise these combinations (t-test on NES values of combination vs. single drugs, p = 0.045; Supplementary Table S3), consistent with a stronger potential effect on HIV suppression.

In addition to *ACTG1*, which was the top downregulated gene by four combination drug regimens yet upregulated by TAF alone, four additional HIV-associated genes (*YBX1*, *TUBB4B*, *ARF1*, and *PPP2R1A*) were downregulated by at least two different ART regimens. Conversely, five single drugs, namely EFV, FTC, TDF, COBI, and RTV, were found to upregulate *ORM2* (Orosomucoid 2) encoding alpha-1-acid glycoprotein 2 (AGP2). Notably, AGP2 is a key acute phase plasma protein that binds HIV envelope glycoprotein and CCR5 which plays a role in HIV infection (Rabehi et al., 1995; Atemezem et al., 2001). Certain single ART drugs such as EFV, DRV, DTG, and TAF each upregulated two or more host-HIV-interacting genes, whereas other single drugs regulated just one. Overall, across different cells, combination ART regimens appeared to target distinct HIV-associated transcriptional targets compared to individual drugs and were more likely to downregulate the expression of genes associated with the viral life cycle.

### ART-inducing disease risk profiles

Although single ART medications are sometimes effective in suppressing viral load temporarily, they carry the potential to cause severe adverse events (Panel on Clinical Practices for Treatment of HIV Infection, 2001), which are often associated with a specific drug or a drug class. Therefore, to evaluate whether combination ART regimens have safer molecular profiles, we cross-referenced tissue-specific transcriptional signatures of drug response to single drugs and combination ART regimens with gene sets associated with lipid profiles, body weight-related traits, and renal function, the most common adverse events of ART, using the GWAS catalog (Sollis et al., 2023) (see *Methods* and Supplementary Table S2).

#### LDL cholesterol

Regimens containing specific ART drugs have been associated with increased dyslipidemia and higher incidence of cardiovascular events (Triant, 2013). To investigate potential genetic mediators, we explored the overlap between the genes associated with LDL levels from GWAS (Supplementary Table S2) and ART-induced differentially expressed genes in HepG2 cells. The transcriptional signatures specific to drugs like EFV, TDF, FTC, RTV and COBI were significantly enriched for LDL-associated GWAS genes (Supplementary Table S4; Supplementary Figure S7). Among combination regimens, only EFV/FTC/TDF showed enrichment for LDL-associated GWAS genes, while ABC/DTG/FTC and DRV/FTC/RTV/TDF showed overall downregulation of LDL-associated genes, suggesting a possible protective effect.

**FIGURE 5:**
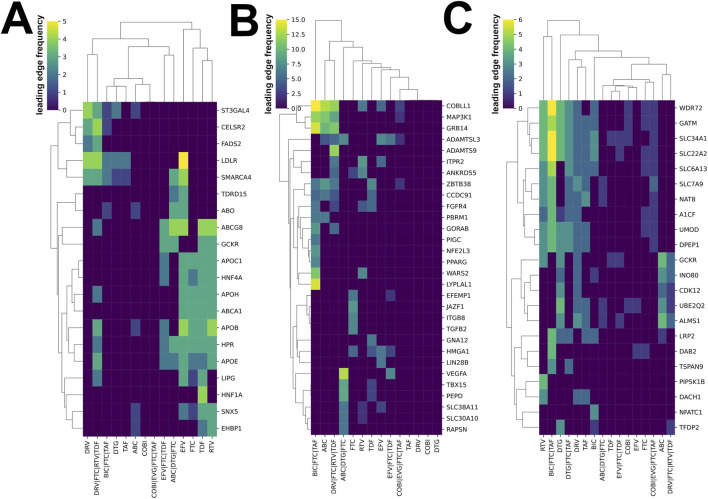
Heatmap of the genes driving the enrichment for adverse effects in the transcriptional responses to antiretroviral therapy (ART). **(A)** Low-density lipoprotein-associated genes and ART responses in the HepG2 cells; **(B)** Body composition-associated genes and ART responses in the HepPG2 cells, and **(C)** Kidney-associated genes and ART responses in the HK2 cells. The number of times each gene appears in the leading edge of the gene set associated with a particular phenotype.

We next sought to identify candidate genes underlying these effects (Figure 5A). EFV-, FTC-, TDF-, and RTV-induced signatures were enriched for *APOB* (Apolipoprotein B)*, APOC1* (Apolipoprotein C1), *ABCA1* (ATP-binding Cassette Transporter A1), *HPR* (Haptoglobin-Related Protein), *APOE* (Apolipoprotein E), and *HNF4A* (Hepatocyte Nuclear Factor 4 Alpha). On the other hand, LDL Receptor (*LDLR,* encoding LDL receptor) was most closely associated with EFV and, to a lesser extent, with DRV, DTG, and TAF, and ART combinations such as DRV/FTC/RTV/TDF and BIC/FTC/TAF. An additional, independent cluster comprising *ST3GAL4*, *CELSR2* and *FADS2* appeared to mediate LDL effects specifically for DRV/FTC/RTV/TDF and DRV. COBI-containing ART signatures showed the weakest enrichment for LDL-associated genes.

To assess whether these effects are hepatocyte specific, we performed parallel analyses in THP-1-derived macrophages. EFV was the only drug whose perturbation signature was enriched for LDL-associated genes, whereas EVG showed significant downregulation (Supplementary Table S3; Supplementary Figure S8). This finding suggest that ART-driven modulation of LDL-related genes is predominantly mediated through liver-specific mechanisms.

#### Body composition

ART regimens have been linked to changes in fat distribution and body composition (Lana et al., 2014; de Waal et al., 2013; Bares et al., 2024; Venter et al., 2019). Accordingly, we compiled GWAS-derived gene sets associated with the adiposity traits, body fat and waist circumference (Supplementary Table S2), and tested their enrichment in HepG2 signatures. Consistent with observational reports, (Venter et al., 2019), ABC and TAF signatures showed significant enrichment of upregulated body fat composition-associated genes, while TDF and FTC showed downregulation (Supplementary Table S4). Several ART regimens exhibited bidirectional patterns across body fat distribution gene sets (Supplementary Figure S9). Candidate mediator analysis showed that most putative driver genes were treatment specific. We identified only one shared cluster of genes across BIC/FTC/TAF, ABC, and DVR/FTC/RTV/TDF, comprising *COBLL1* (Cordon-Bleu WH2 Repeat Protein Like 1), *GRB14* (Growth Factor Receptor Bound Protein 14), and *MAP3K1* (Mitogen-Activated Protein Kinase 1). *LYPLAL1* (Lysophospholipase Like 1) was most strongly associated with BIC/FTC/TAF, whereas *VEGFA* (Vascular Endothelial Growth Factor A) with ABC/DTG/FTC (Figure 5B).

#### Renal function

ART can negatively affect kidney function through distinct mechanisms ranging from proximal tubular toxicity (e.g., TDF-induced toxicity), to benign increase in serum creatinine due to inhibition of tubular transporters and reduced tubular creatinine secretion (e.g., DTG, COBI, RTV, and BIC) (Milburn et al., 2017). We compiled a GWAS-derived gene set associated with estimated glomerular filtration rate (eGFR; Supplementary Table S2) and tested whether these genes significantly overlap with different ART transcriptional signatures in the proximal tubular HK-2 cell line. eGFR-associated genes were significantly downregulated in 5 out of 9 single ART signatures, — particularly following COBI, RTV or BIC therapies, which are known to inhibit tubular secretion—as well as TAF and DRV, and in 3 out of 6 ART combination regimens, each containing at least one single ART linked to tubular dysfunction (Supplementary Table S4; Supplementary Figure S10). In contrast, FTC and DTG signatures showed upregulation of eGFR-associated genes. We identified two recurrent clusters of driver genes. One cluster (*SLC7A9*, *SLC6A13*, *SLC34A1*, *SLC22A2*, *WDR72*, *GATM*, *NAT8*, *A1CF*, *DPEP1*, *LRP2*, and *UMOD)* was shared across RTV, BIC/FTC/TAF, DTG, DTG/FTC/TAF, DRV, and TAF signatures (Figure 5C), whereas the second cluster (*GCKR*, *INO80*, *CDK12*, *UBE2Q2*, and *ALMS1*) recurred across ABC, DTG, and DTG/FTC/RTV/TDF.

### ART signature portal utility

The *ARTexpress* signature portal enables users to browse all pre- and post-treatment gene activity data across three cell lines (HepG2, HK-2, THP-1) and 44 ART regimens (11 single agents, 20 two-way, 11 three-way, and 2 four-way combinations). Users can also browse by regimen and cell line, select specific time points, and view the correspondent transcriptional signatures summarized as modulated z-score gene activity profiles. The portal ARTexpress also performs enrichment analysis for single gene sets and paired up/down gene sets using blitzGSEA, reporting NES, composite scores for paired sets, and directionality labels (mimicker, reverser, ambiguous). All signatures, gene activity profiles, and tissue-specific gene networks can be downloaded for offline analysis.

## Discussion

This study applied high-throughput transcriptomics to analyze how human cell lines representative of liver (HepG2), kidney (HK-2) and innate immune cells (THP-1) respond to real-world ART regimens, both as individual drugs and clinically relevant combinations. Across tissues, ART combination regimens produced transcriptional patterns that were distinct from each single drug in the combinations. Combinations more strongly perturbed genes encoding proteins involved in HIV-host interactions and were less tightly coupled to signatures associated with common ART-related side effects, potentially explaining their higher efficacy and lower toxicity profiles. We also identified genes and pathways consistently targeted by ART, offering new leads for ART development.

We found that *ACTG1* was downregulated across four ART combination regimens, whereas other genes showed regimen specificity. *ACTG1* encodes for gamma-actin, a core cytoskeleton component that supports HIV viral entry, intracellular trafficking, and virion (Warrilow and Harrich, 2007; Cabrera-Rodriguez et al., 2023; Ospina Stella and Turville, 2018). Consistent with prior reports that disrupting actin polymerization reduces HIV infectivity (Iyengar et al., 1998; Aggarwal et al., 2017), *ACTG1* was previously found to be downregulated in ART naïve PWH who naturally control HIV-1 replication to some extent preventing or delaying clinical progression to AIDS (Lee et al., 2019), suggesting its role as a candidate host mediator. Additional putative molecular targets included *YBX1*, *PPP2R1A*, and *ARF1*, which were downregulated by two distinct ART regimens*. YBX1*, which encodes the Y-box-binding protein 1, supports early and late steps of HIV replication (Weydert et al., 2018); *PPP2R1A*, encoding the alpha (α) subunit of Protein Phosphatase 2A (PP2A), contributes to cell growth, division, and signal transduction pathways, and interacts with the HIV-1 protein Vpr (Barski et al., 2021); and *ARF1* (encoding ADP-ribosylation factor 1) facilitates HIV immune evasion (Morris et al., 2018). Notably, the corresponding single drugs did not significantly affect these genes, suggesting low-threshold synergistic off-target effects emerging only in combination. In contrast, five single drugs—EFV, FTC, TDF, COBI, and RTV—upregulated ORM2, an acute-phase protein that modulates metabolic and inflammatory responses (Heo et al., 2024) and reportedly blocks HIV entry *in-vitro* (Bosinger et al., 2004). Because HIV replication can suppress *ORM2* (Seddiki et al., 1997), its upregulation may reflect reduced viral effects. Importantly, ORM2 also rises under inflammatory, metabolic, or drug-induced stress, suggesting an increased general cellular or liver strain following ART monotherapy. This effect was absent in combination ART, likely due to lower drug levels and broader adaptive pathways that limit stress. Finally, *EEF1G* and *EEF1B2,* encoding subunits of eukaryotic elongation factor 1, were downregulated exclusively with DTG/FTC/TAF regimen. Given that eEF1 subunits stabilize HIV-1 reverse transcription complex and facilitate the of viral DNA synthesis (Warren et al., 2012; Li et al., 2015), this pattern is consistent with the inclusion of the NRTIs FTC and TAF in that regimen.

We next investigated whether ART-induced transcriptional responses recapitulate known adverse-effect profiles related to dyslipidemia, body fat distribution, or renal dysfunction, previously linked to older generation ART medications (Kalra et al., 2023; Thet and Siritientong, 2020; Post, 2014). In hepatocytes, EFV, FTC, RTV and COBI signatures were enriched for upregulated LDL-associated genes, aligning with prior observational studies of elevated lipid levels associated with these agents (Panel on Clinical Practices for Treatment of HIV Infection, 2001; Molina et al., 2011). No significant enrichment was observed for ABC, DRV, or DTG, which are known to have a relatively better lipid profile in clinical settings (Podzamczer et al., 2007; Saumoy et al., 2021; Echeverria et al., 2017). We also observed enrichment for LDL-associated genes following TDF treatment, but not with TAF, which contrasts clinical reports that TAF increases LDL-cholesterol levels (Panel on Clinical Practices for Treatment of HIV Infection, 2001; Brunet et al., 2021) and that switching from TDF to TAF worsens lipid profiles, irrespective of pharmaco-enhancer or third-agent use (Brunet et al., 2021).

These effects on lipid metabolism genes were predominantly found in hepatic cells as shown by a weaker or no enrichment in THP-1 cells. Two sets of candidate lipid metabolism driver genes emerged in the liver: EFV, FTC, TDF, and RTV engaged *APOB*, a primary component of LDL (Galimberti et al., 2023), and *HNF4A*, a liver transcription factor regulating lipid and glucose metabolism (Hayhurst et al., 2001). This effect of ART on lipid metabolism is consistent with prior evidence that RTV can modulate HNF4A (e.g., via non-coding RNAs) (Wang et al., 2022; Gwag et al., 2019), and that ART and HIV infection itself (Mujawar et al., 2006) can both inhibit *ABCA1,* critical in cholesterol efflux (Brewer et al., 2004), potentially increasing cardiovascular risk associated with of dyslipidemia. A second group associated with DRV/FTC/RTV/TDF and DRV therapies comprised three driver genes: *ST3GAL4,* implicated in inflammatory leukocyte recruitment and atherosclerosis progression in mouse models (Doring et al., 2014), *CELSR2,* which modulates hepatic lipoprotein handling (Tan et al., 2021), and *FADS2,* whose dysregulation can lead to altered blood lipid levels in children (Standl et al., 2012) and lipid accumulation in the murine liver (Hayashi et al., 2021).

ART-related changes in body weight and fat distribution are well documented, including weight gain and conditions such as lipoatrophy and lipodystrophy (Lana et al., 2014; de Waal et al., 2013; Kanters et al., 2022). Here, in HepG2 cells, TAF treatment upregulated weight and body fat distribution-related genes, with a milder association also observed for other TAF-containing regimens such as COBI/EVG/FTC/TAF and BIC/FTC/TAF. This finding is supported by previous studies demonstrating that some PWH who switched to BIC/FTC/TAF experienced weight gain (Perez-Barragan et al., 2023). On the other hand, the weight and body fat distribution-related genes were downregulated by TDF, mirroring clinical data of weight gain with TAF (Panel on Clinical Practices for Treatment of HIV Infection, 2001; Venter et al., 2019; Mallon et al., 2021; Sax et al., 2020), and relative weight neutrality of weight loss with TDF or the TDF/FTC combination (Baeten et al., 2012). These off-target effects could be linked to several driver genes in a treatment-specific manner. For example, ABC, DRV/FTC/RTV/TDF and BIC/FTC/TAF seemed to upregulate *GRB14*, which is involved in insulin signaling and the regulation of metabolic pathways (Carre et al., 2008), and may promote adipogenesis and influence fat storage in adipocytes. *MAP3K1* has likewise been implicated in regulating adipocyte differentiation and lipid storage (Brunet et al., 2021). Conversely, EFV/FTC/TDF transcriptional signature was associated with reduced activity of body composition-related genes, consistent with the observation that TDF-containing treatment regimens are generally associated with less weight gain (Sax et al., 2020) and that switching from EVR-containing regimens can ameliorate lipoatrophy (Rojas et al., 2016). FTC and its combination DRV/FTC/RTV/TDF showed a similar downregulation of body composition-associated genes, even when FTC was combined with TDF.

To investigate potential renal off-target effects of ART, we analyzed the transcriptional activity of genes associated with estimated glomerular filtration rate (eGFR) in the HK-2 cell line. We observed significant downregulation of eGFR-associated genes for several single ART drugs, including COBI, BIC, RTV, DRV, and TAF. This is consistent with experimental evidence that COBI (Lepist et al., 2014), BIC, RTV, and DTG (Nakayama et al., 2024; Rivero and Domingo, 2015) impact serum creatinine levels by inhibiting the renal tubular transporter SLC22A2 (OCT2), reducing creatinine secretion without renal damage (Gutierrez et al., 2014). Combinations containing these ART drugs—DTG/FTC/TAF, BIC/FTC/TAF, and COBI/EVG/FTC/TAF—showed similar physiological regulation of eGFR-associated gene activity, in line with safe clinical kidney function profile (Surial et al., 2020; Eron et al., 2024; Bonora et al., 2016; Post et al., 2017). *SLC22A2* (*OCT2*) and other renal transporters appeared among the kidney driver genes shared by BIC, DTG, RTV, DRV, TAF, and the above combinations. In contrast, TDF, a drug with established tubular nephrotoxicity (Post, 2014), did not show enrichment for eGFR-associated genes in our analysis.

Of note, combination ART regimens that include pharmacokinetic enhancers such as RTV and COBI may interact with co-medications, particularly through their effects on cytochrome P450 enzymes and drug transporters (Marzolini et al., 2016). However, further experimental validation would be necessary to determine their contribution to the observed renal, metabolic, and weight-related adverse effects, and to disentangle direct drug effects from those mediated by altered exposure to concomitant medications.

The key strength of this study is the focus on molecular responses to real-world first-line HIV therapies, which are directly relevant to current clinical practice. We used three established cell line models: THP-1 for foam cell formation, a hallmark of atherosclerosis; HK-2, for investigating renal dysfunction and nephrotoxicity; and HepG2 for modeling liver metabolism and hepatotoxicity. While cell lines offer distinct advantages, including ease of use and ability to model cellular and pathological processes, they remain limited by their artificial nature, such as genetic drift, altered physiology, and loss of tissue-specific properties, and cannot fully replicate human disease complexity. Additional limitations include the use of the L1000 transcriptomics assay, which directly measures 978 landmark genes while inferring the remainder computationally. Although L1000 is not a full-transcriptome platform, it is well suited to capture robust perturbational signals. Yet, because only a subset of genes is directly assayed, tissue-specific changes may be underrepresented. To emphasize reproducible effects, we pooled transcriptional signatures across cell lines, focusing on concordant directionality to highlight shared drug responsive genes and pathways while reducing cell type-specific noise. Moreover, our analysis reflects acute exposure windows, and longer-term treatment may elicit additional adaptations that were not assessed here. Finally, experiments were performed in uninfected cell lines; responses in the context of HIV infection may differ, and extending these analyses to infected systems and longer exposures is a natural next step.

In summary, combination ART regimens elicited transcriptional responses that were distinct from those induced by single ART drugs. Combination-induced gene expression signatures were significantly more enriched for genes involved in HIV-host protein-protein interactions and were less tightly coupled to signatures associated with common ART-related side effects. These findings underscore the value of high-throughput transcriptomics for delineating off-target mechanisms and guiding the development of safer, more effective ART regimens. Furthermore, *ARTexpress* makes these data accessible and reusable, facilitating dissemination and further discovery by the research community.

## Data Availability

The datasets presented in this study can be found in online repositories. The data have been deposited to Gene Expression Omnibus (GEO) on 20 December 2025; the GEO accession number is GSE314659. L1000 data and processed drug perturbation signatures are also available for download from the ARTexpress website at https://maayanlab.cloud/artexpress.
