# Supplementary material for: Transcriptional response to combination antiretroviral therapy predicts side effects and novel targets
Source: Front Pharmacol. 2026 Jan 21;16:1743543. doi: 10.3389/fphar.2025.1743543 (PMC12868293; doi:10.3389/fphar.2025.1743543)
Supplement: Supplementary file 1 [file Supplementaryfile1.docx]

**Supplemental Material**

**Transcriptional Response to Combination Antiretroviral Therapy Predicts Side Effects and Novel Targets**

Alexander Lachmann, Letizia Amadori, Paola Nicoletti, Heidi M. Crane, Chiara Giannarelli, Avi Ma’ayan, and Inga Peter

**Contents**

[**Supplemental Methods** 2](#_Toc216360829)

[**Figure S1**: Sample reproducibility of positive and negative controls for touchstone perturbations. 4](#_Toc216360830)

[**Figure S2**: Bar plot depicting the ranking of drug perturbations based on signature strength. 5](#_Toc216360831)

[**Figure S3**: Signature strength of touchstone drugs relative to ART drug perturbation signature strengths. 6](#_Toc216360832)

[**Figure S4**: Quality control analysis of drug expression signatures compared to characteristic gene expression signatures and corresponding ARCHS4 gene expression data. 7](#_Toc216360833)

[**Figure S5**: Plate-drug self-similarity by cell line and treatment duration 8](#_Toc216360834)

[**Figure S6:** UMAP of consensus gene activity signatures of ART drugs and drug combinations 9](#_Toc216360835)

[**Figure S7**: GWAS enrichment for low density lipoprotein in HEPG2 cells 10](#_Toc216360836)

[**Figure S8**: GWAS enrichment for low density lipoprotein in THP-1 cells 11](#_Toc216360837)

[**Figure S9**: GWAS enrichment for body weight-related traits in HEPG2 cells 12](#_Toc216360838)

[**Figure S10**: GWAS enrichment for kidney-related phenotypes in HK2 cells 13](#_Toc216360839)

[**Table S1**: ART treatment dosage 14](#_Toc216360840)

[**Table S2**: The list of gene set terms used in genome-wide association studies. 15](#_Toc216360841)

[**Table S3**: Enrichment of the host-HIV interaction genes among ART transcriptional signatures across the cell lines. 16](#_Toc216360842)

[**Table S4**: GWAS enrichment for lipid profile, body weight-related traits, and kidney phenotype by ART regimen in different cell types. 17](#_Toc216360843)

[**References** 18](#_Toc216360844)

# **Supplemental Methods**

*Drug Dose Selection*

The dose selection was based on the conversion from human equivalent dose used in clinical settings to dosages applicable to in vitro settings, using the body surface area normalization method^1^. Subsequently, a titration curve was designed, and each drug was tested for viability, cytotoxicity and apoptosis/necrosis. The final concentration was selected based on both the in vitro testing results and by referring to dosages previously used in the same cell line settings^2-18^.

*Sample Reproducibility*

To measure the reproducibility of gene expression profiles across replicate samples, we assessed each plate individually. Each plate consisted of a gene expression matrix with 12,328 genes (rows) and 306 to 320 samples (columns), depending on experimental conditions. From an initial set of 324 wells per plate, low-quality wells were automatically filtered out using L1000 sample filtering criteria, based on internal quality control measures such as low bead counts. For each combination of plate and drug, we retrieved the replicate samples corresponding to that drug and calculated pairwise Pearson correlation coefficients between their gene expression profiles. To exclude self-correlations, the diagonal elements of the correlation matrix were set to NaN. The average of the remaining off-diagonal correlation values was then computed, providing a measure of average reproducibility for that drug perturbation on that plate (**Figure S1**). This approach ensured consistent evaluation of reproducibility by comparing gene expression patterns across quality-filtered replicate samples.

*Touchstone Perturbation Validation and Signature Strength*

To evaluate the consistency and strength of drug perturbation signatures, we analyzed touchstone drugs known for their robust and reproducible differential gene expression profiles: Vorinostat (a histone deacetylase inhibitor), Rapamycin (an mTOR inhibitor), Wortmannin (a phosphoinositide 3-kinase inhibitor), and Genistein (a protein tyrosine kinase inhibitor). For each drug perturbation, the signature was defined as the set of differentially expressed genes, and signature strength was calculated as the sum of the absolute values of differential gene expression for the genes within the signature. This computation was performed separately for signatures composed solely of landmark genes and for those including inferred genes. Gene expression profiles were filtered to exclude low-quality samples, after which signature strength was calculated for all drug perturbation signatures across six plates. For each plate, all signatures were ranked based on their strength. The ranks of the touchstone signatures were extracted from each plate and combined into a single list representing the ranks across all six plates. Using this combined ranking, we computed the area under the curve (AUC) to assess whether touchstone signatures were consistently ranked highly. To evaluate the statistical significance of the observed AUC, we conducted a permutation test. The rank order was shuffled 100,000 times, and the AUC was recalculated for each permutation to generate a distribution of randomized AUCs. A normal distribution was estimated from the mean and standard deviation of these randomized AUCs, and the cumulative distribution function was used to compute a p-value for the observed AUC (**Figures S4-S5**).

*ARTexpress Signature Portal*

The ARTexpress signature portal was developed to enable gene set enrichment analysis using precomputed drug perturbation signatures. Signature data are stored in AWS S3 in pickle format and loaded into memory upon server initialization. The portal supports two enrichment analysis modes: single gene set enrichment and paired up/down gene set enrichment. Enrichment calculations were performed using BlitzGSEA, a rapid gene set enrichment analysis algorithm, with the following parameters: a shared null distribution with a Kullback-Leibler divergence threshold of 200, computed from 500 permutations using 10 anchor points, and a maximum gene set size of 1000. For paired gene sets, enrichment was calculated separately for the up-regulated and down-regulated gene sets. The normalized enrichment scores (NES) for each were multiplied to derive a composite enrichment score, which is always positive. The portal is implemented using FastAPI and deployed as a Docker container with an Ubuntu 22.04 base image on a Kubernetes cluster managed by Rancher orchestration. The source code is publicly available at <https://github.com/MaayanLab/artexpress>.

# **Figure S1**: Sample reproducibility of positive and negative controls for touchstone perturbations.


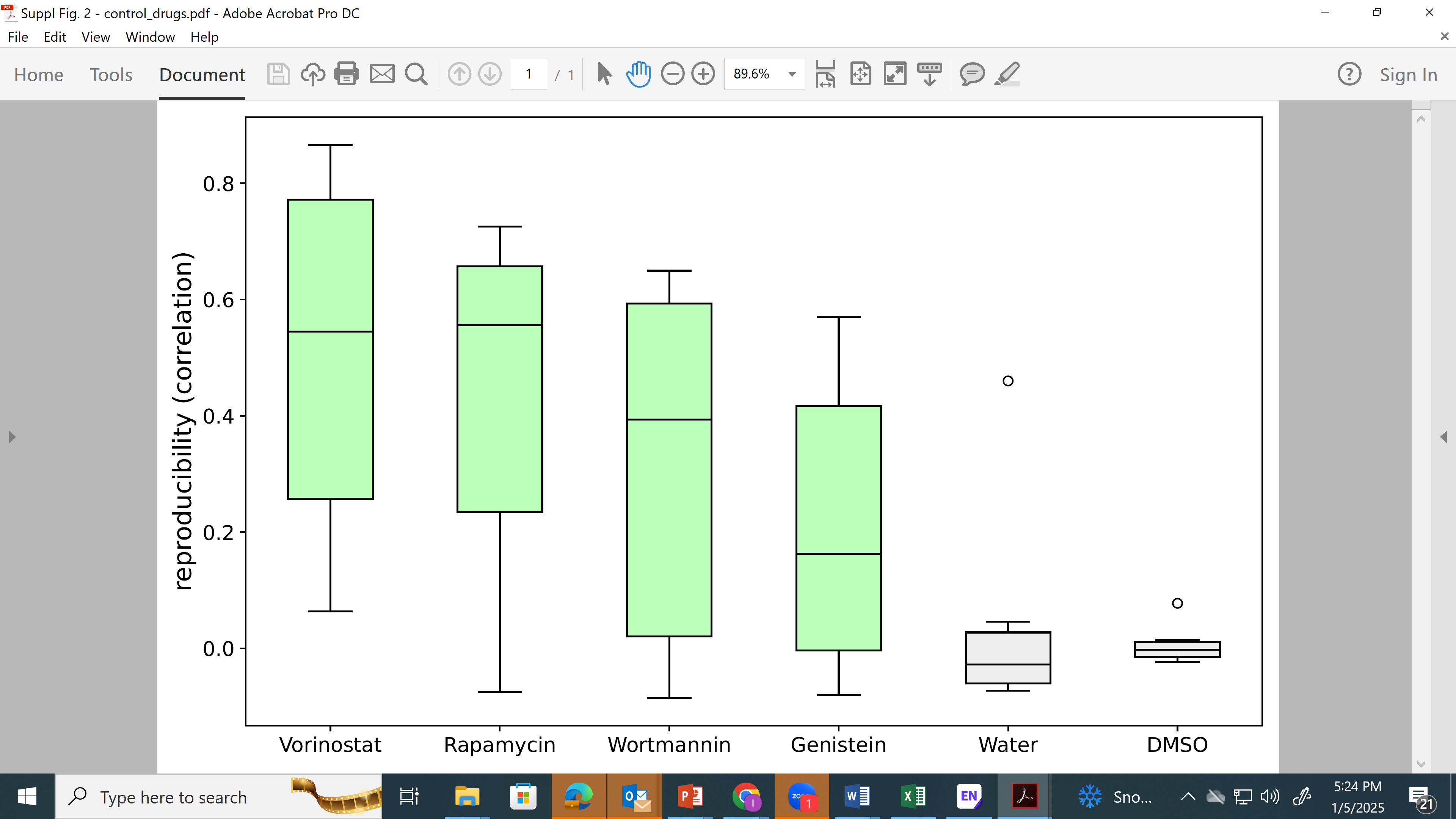


Touchstone drug perturbations with known strong reproducibility in replicates (Vorinostat, Rapamycin, Wortmannin, Genistein) and perturbations with low reproducibility (Water, DMSO). The plot shows correlation of MODZ signatures between replicates on the same plate for touchstone perturbations across plates.

# **Figure S2**: Bar plot depicting the ranking of drug perturbations based on signature strength.


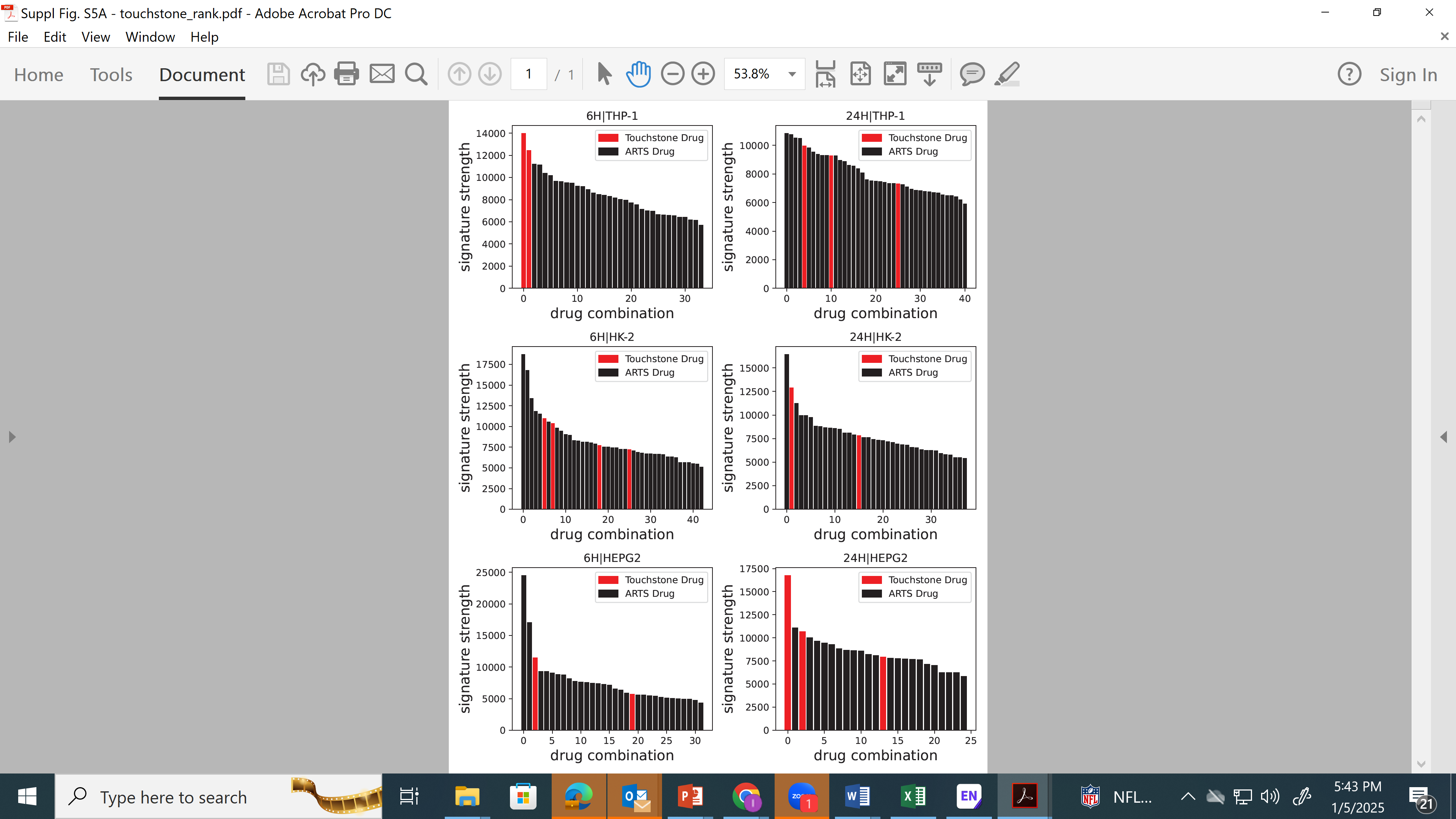


The ranking of drug perturbations based on signature strength was calculated as the sum of absolute modulated z-score values. Touchstone drugs (Vorinostat, Rapamycin, Wortmannin, and Genistein), if passing QC, are highlighted in red.

# **Figure S3**: Signature strength of touchstone drugs relative to ART drug perturbation signature strengths.


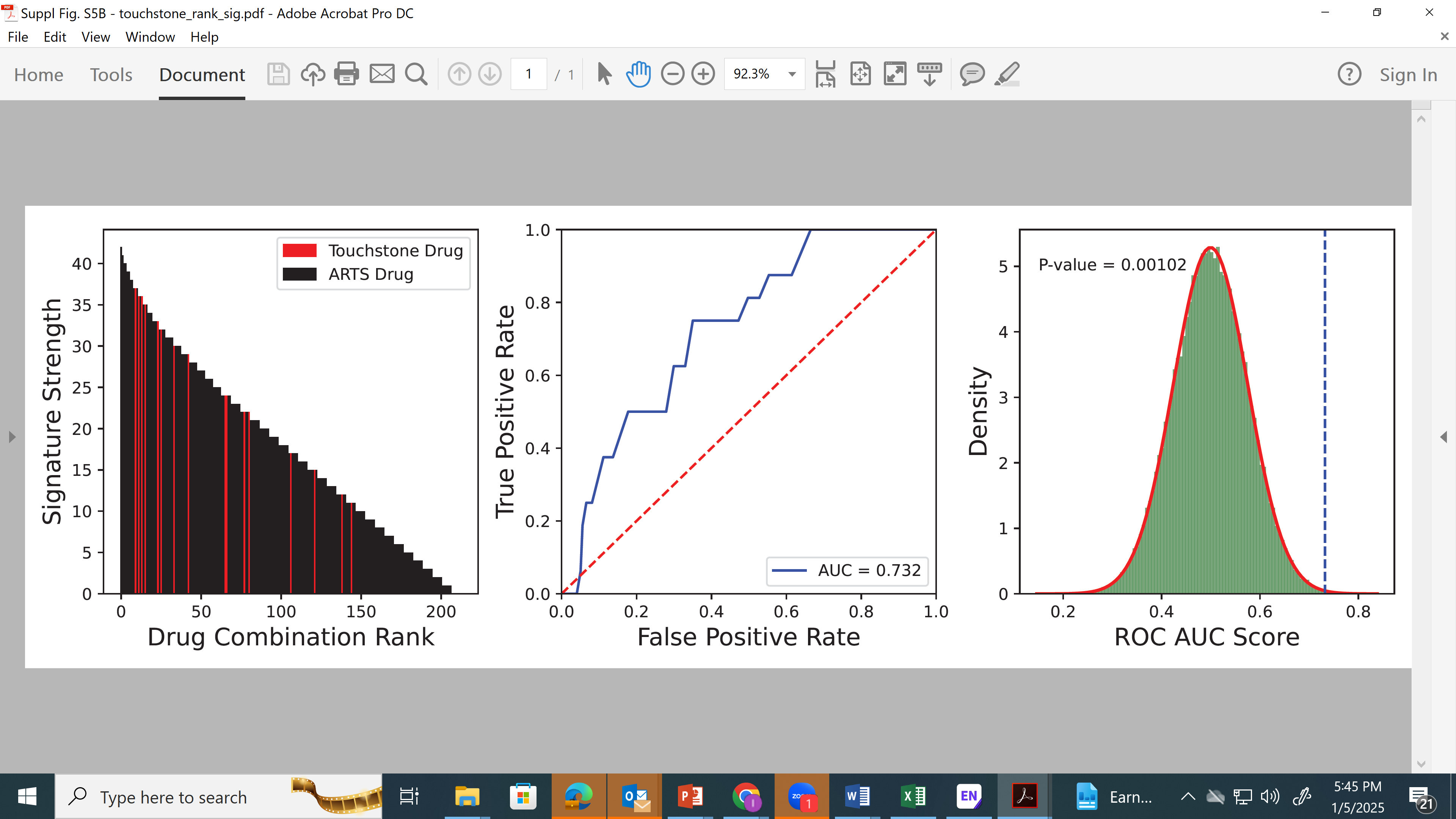


**a**

**b**

**c**

a) Signature strength ranking of all drug perturbations across all plates, with touchstone drugs (Vorinostat, Rapamycin, Wortmannin, and Genistein) highlighted in red. Signature strength was calculated as the sum of absolute modulated z-score values. b) Area under the curve (AUC) of the rank positions of touchstone drugs compared to ART drug treatments. c) Randomized signature strength ranking of touchstone drugs, showing the expected random AUC derived from 10,000 randomized rankings alongside the observed AUC.

# **Figure S4**: Quality control analysis of drug expression signatures compared to characteristic gene expression signatures and corresponding ARCHS4 gene expression data.


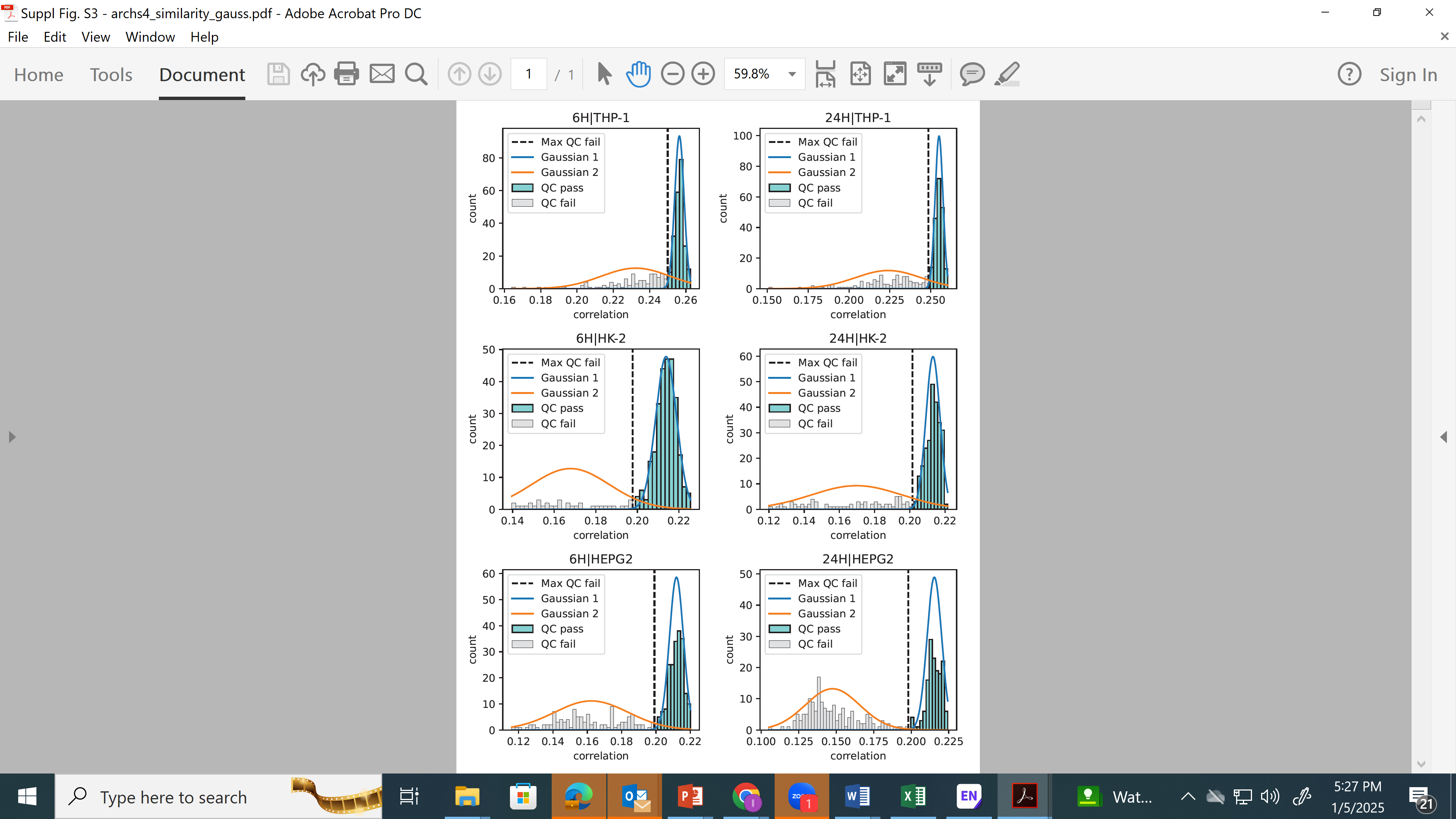


Outliers were identified using a bimodal Gaussian mixture model. Samples assigned to the Gaussian distribution with the lower mean correlation were excluded from further analysis.

# **Figure S5**: Plate-drug self-similarity by cell line and treatment duration


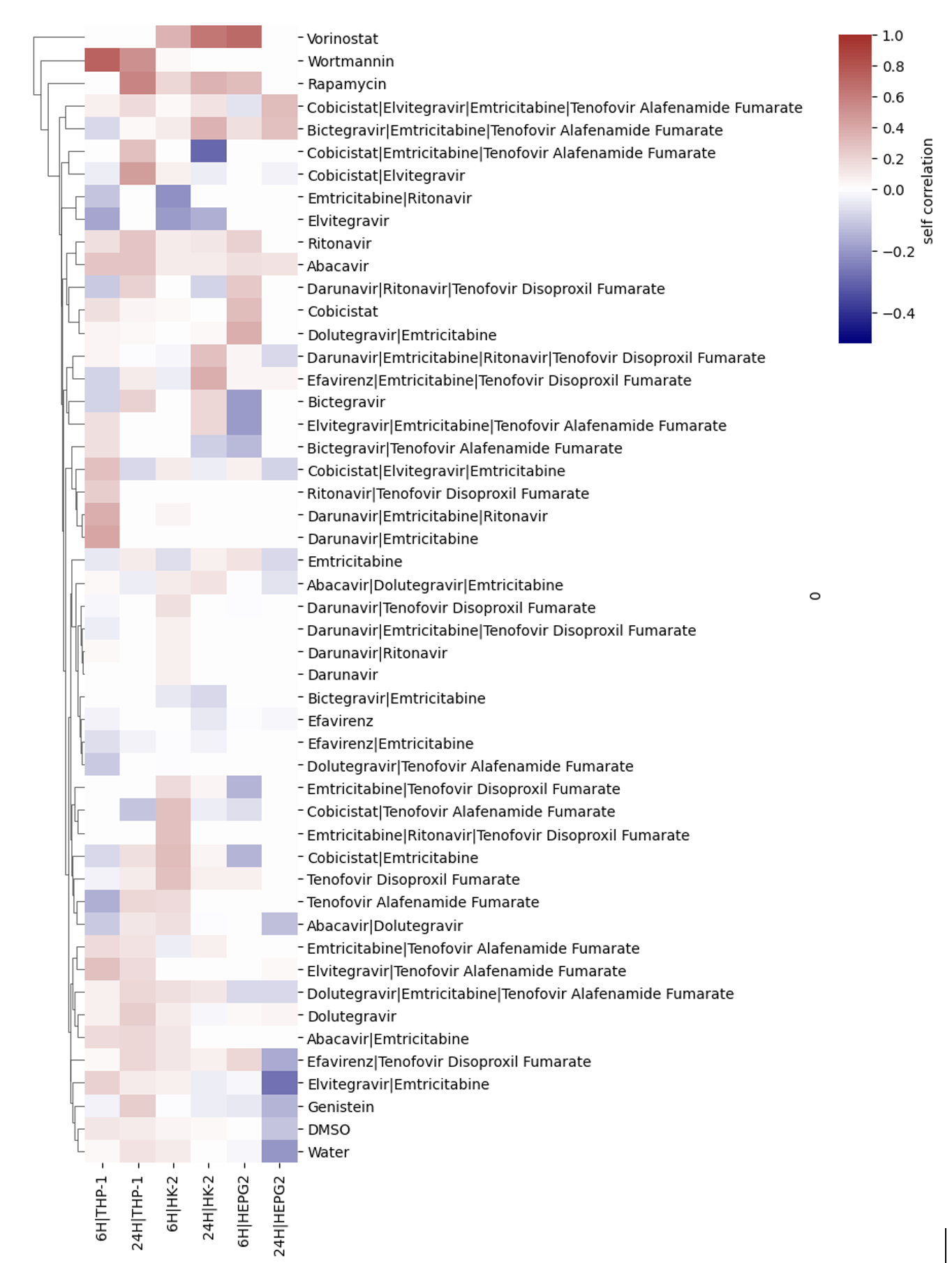


Heatmap displaying self-correlation of replicate samples from the same plate. Each plate contains up to three replicates. Pearson correlation coefficients were calculated to assess similarity between replicates.

# **Figure S6:** UMAP of consensus gene activity signatures of ART drugs and drug combinations


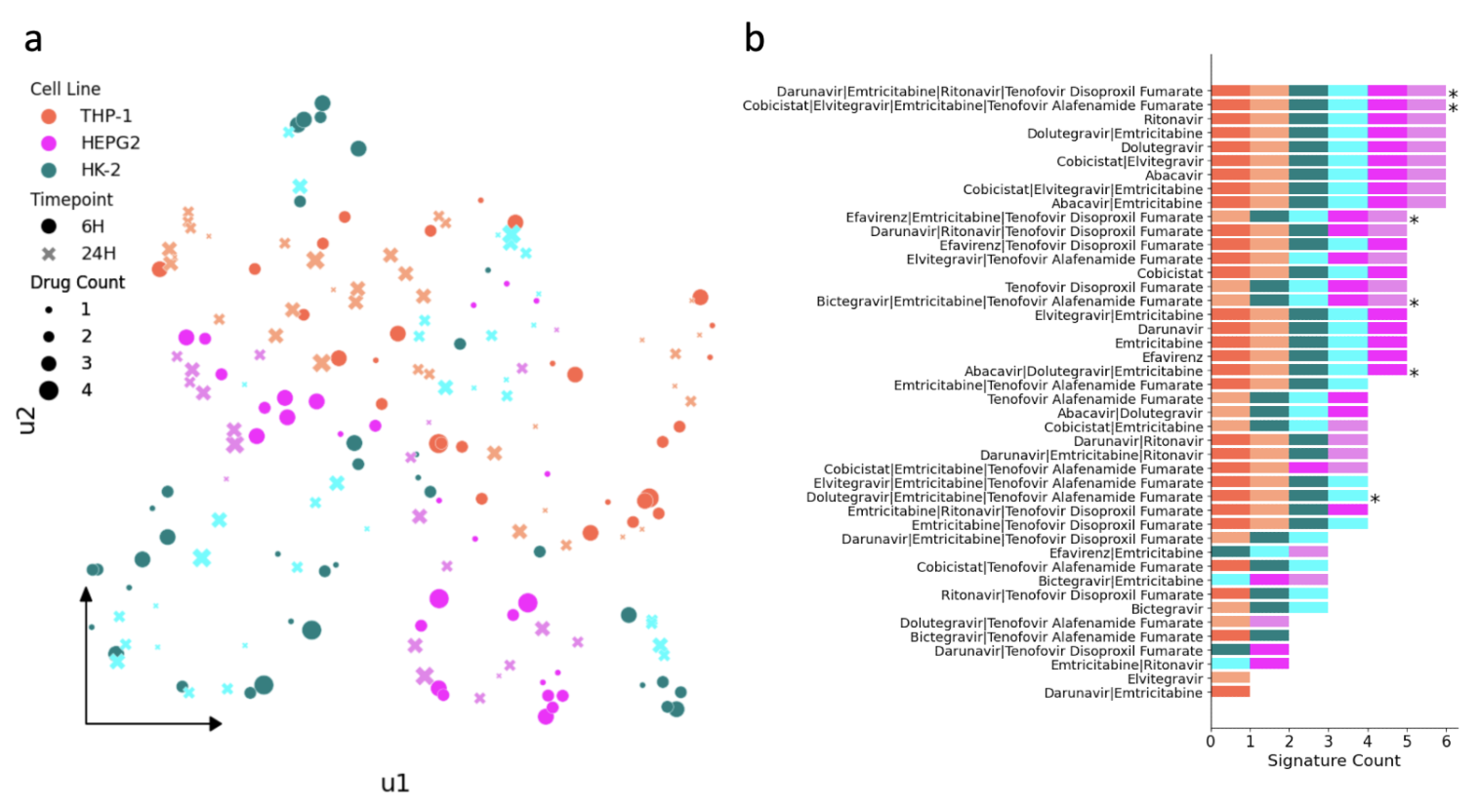


a) UMAP of L1000 drug treatment cell line specific activity profiles for cell lines THP-1, HEPG2, and HK-2 at 6H and 24H time points. b) Drug combination treatment signatures after quality control and their availability under experimental conditions (cell line and time point). *indicates combination ART regimens.

# **Figure S7**: GWAS enrichment for low density lipoprotein in HEPG2 cells


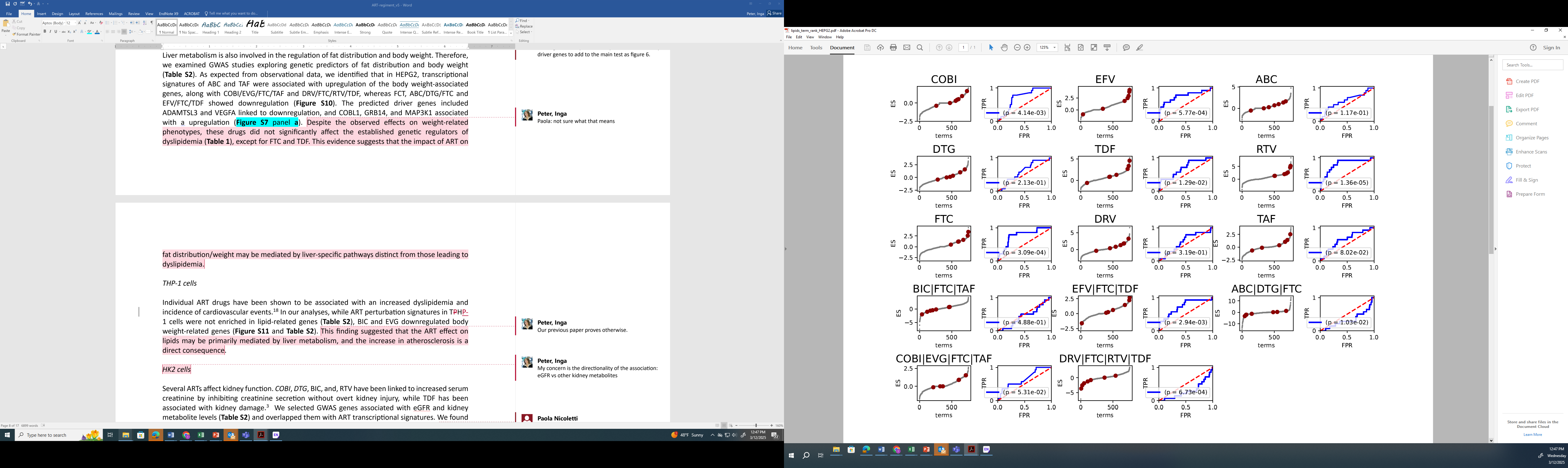


Left: Distribution of enrichment scores for gene sets derived from GWAS summary statistics, calculated from HEPG2 cell responses to single antiretroviral therapy (ART) drugs and their combinations. Right: Area under the curve (AUC) plot with an associated p-value, where the dotted red line represents the expected AUC under a random distribution. P-values are not adjusted for multiple testing. See Table S4 for false discovery rate-adjusted P-values.

# **Figure S8**: GWAS enrichment for low density lipoprotein in THP-1 cells


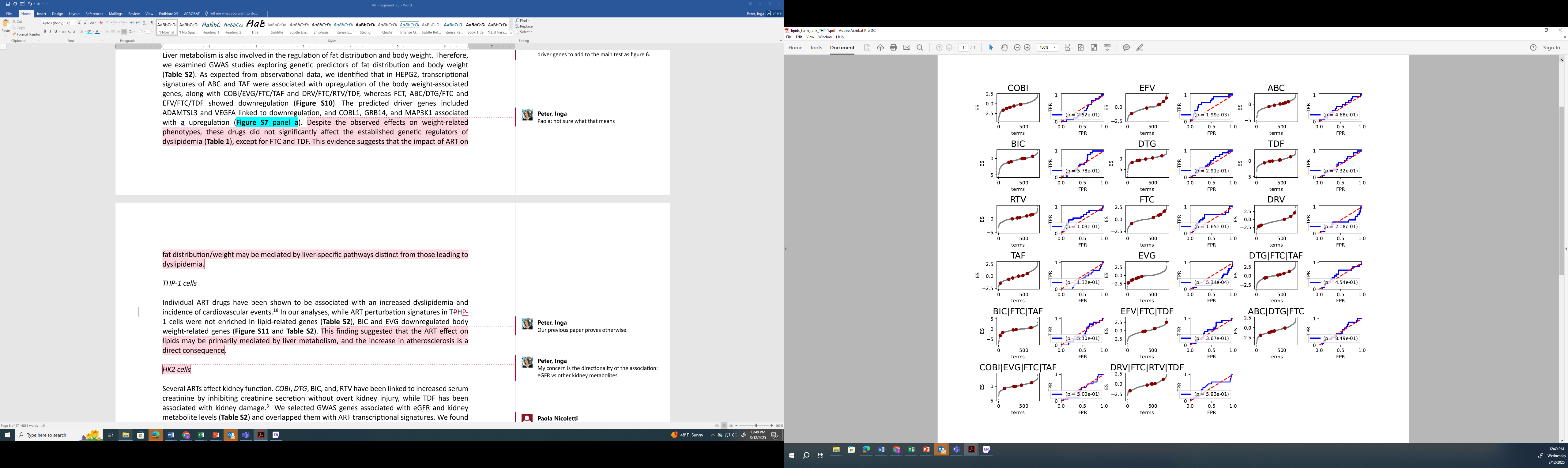


Left: Distribution of enrichment scores for gene sets derived from GWAS summary statistics, calculated from THP-1 cell responses to single antiretroviral therapy (ART) drugs and their combinations. Right: Area under the curve (AUC) plot with an associated p-value, where the dotted red line represents the expected AUC under a random distribution. P-values are not adjusted for multiple testing. See Table S4 for false discovery rate-adjusted P-values.

# **Figure S9**: GWAS enrichment for body weight-related traits in HEPG2 cells


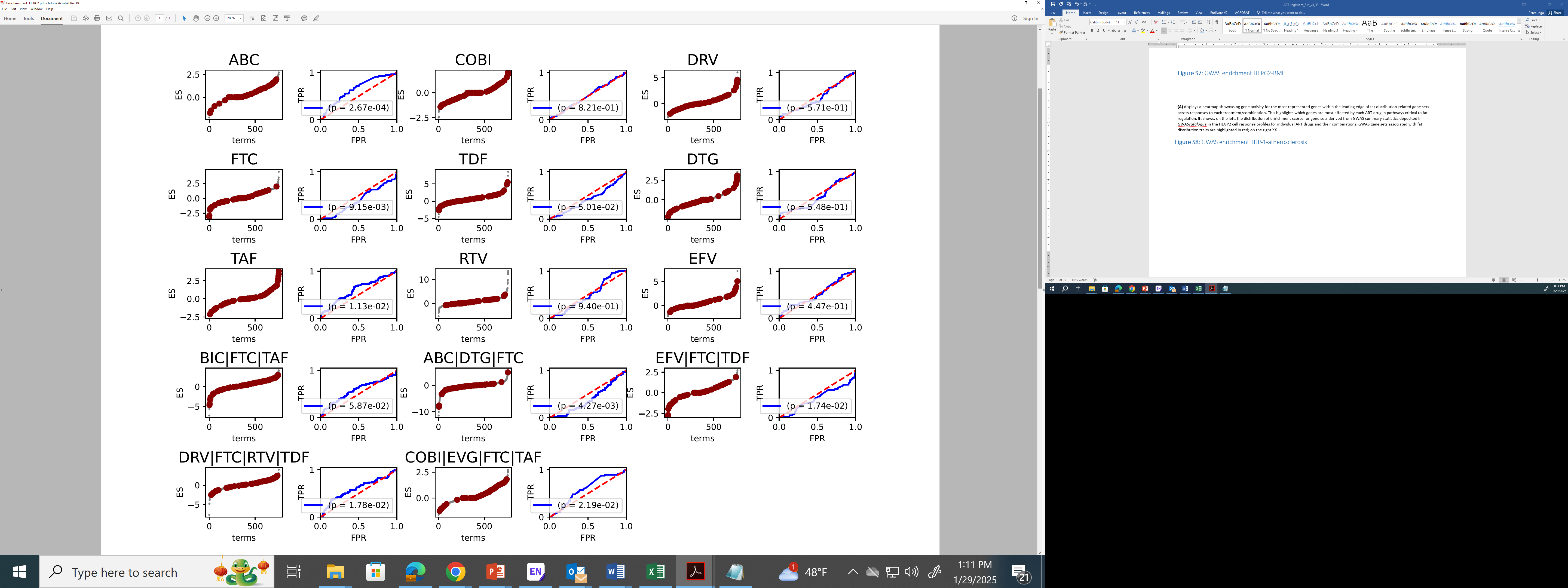


Left: Distribution of enrichment scores for gene sets derived from GWAS summary statistics, calculated from HEPG2 cell responses to single antiretroviral therapy (ART) drugs and their combinations. Right: Area under the curve (AUC) plot with an associated p-value, where the dotted red line represents the expected AUC under a random distribution. P-values are not adjusted for multiple testing. See Table S4 for false discovery rate-adjusted P-values.

# **Figure S10**: GWAS enrichment for kidney-related phenotypes in HK2 cells


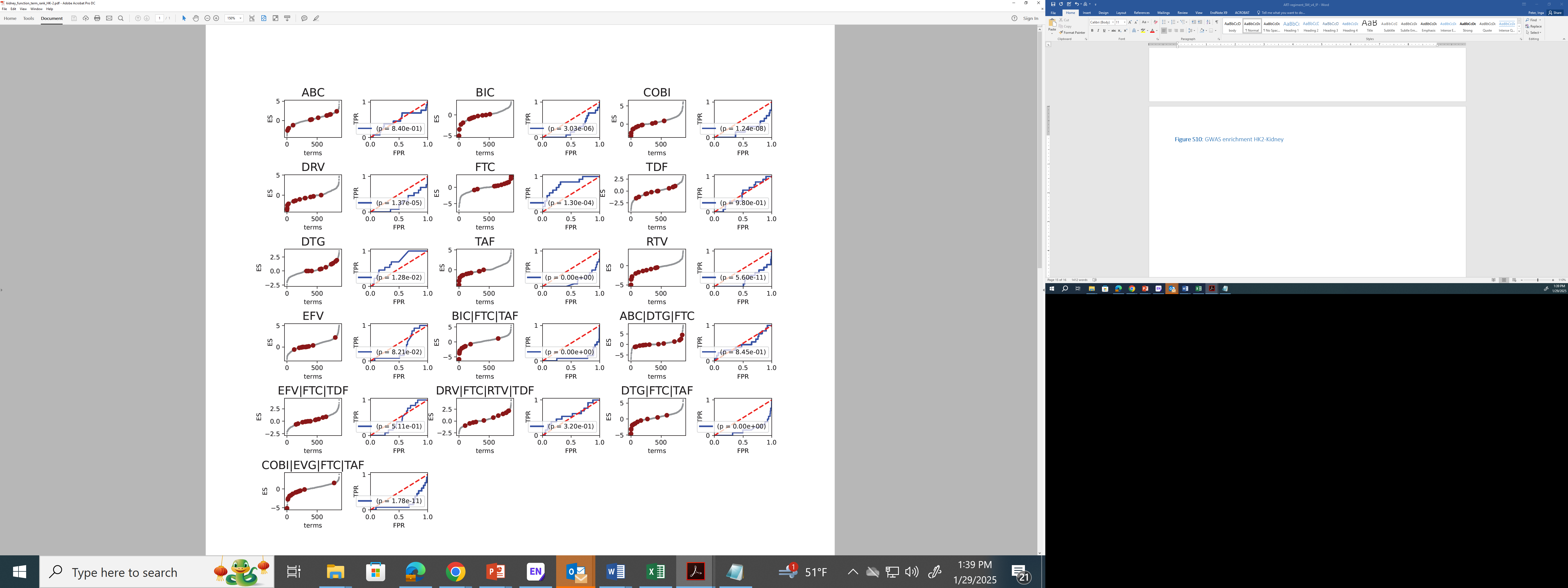


Left: Distribution of enrichment scores for gene sets derived from GWAS summary statistics, calculated from HK2 cell responses to single antiretroviral therapy (ART) drugs and their combinations. Right: Area under the curve (AUC) plot with an associated p-value, where the dotted red line represents the expected AUC under a random distribution. P-values are not adjusted for multiple testing. See Table S4 for false discovery rate-adjusted P-values.

# **Table S1**: ART treatment dosage

| **Compound** | **Cat. No.** | **Company** | **Final concentration (µm)** |
| --- | --- | --- | --- |
| Abacavir sulfate (ABC) | SML0089 | Sigma aldrich | 0.5 |
| Bictegravir (BIC) | A363683 | Ambeed | 0.5 |
| Cobicistat (COBI) | R16030 | Advanced Chemblocks | 0.1 |
| Darunavir (DRV) | SML0937 | Sigma aldrich | 0.1 |
| Dolutegravir (DTG) | 10313 | Advanced Chemblocks | 0.1 |
| Efavirenz (EFV) | SML0536 | Sigma aldrich | 0.5 |
| Elvitegravir (EVG) | S2001 | Selleckchem | 0.1 |
| Emtricitabine (FTC) | PHR 2120 | Sigma aldrich | 0.1 |
| Ritonavir (RTV) | SML0491 | Sigma aldrich | 0.5 |
| Tenofovir alafenamide fumarate (TAF) | M22552 | Advanced Chemblocks | 0.5 |
| Tenofovir disoproxil fumarate (TDF) | SML1794 | Sigma aldrich | 0.1 |
| ABC/DTG/FTC |  |  | 0.5/0.1/0.1 |
| BIC/FTC/TAF |  |  | 0.5/0.1/0.5 |
| COBI/EVG/FTC/TAF |  |  | 0.1/0.1/0.1/0.5 |
| DRV/FTC/RTV/TDF |  |  | 0.1/0.1/0.5/0.1 |
| EFV/FTC/TDF |  |  | 0.5/0.1/0.1 |
| FTC/DTG/TAF |  |  | 0.1/0.1/0.5 |

ABC, Abacavir; BIC, Bictegravir; DTG, Dolutegravir; EFV, Efavirez; RTV, Ritonavir; TAF, Tenofovir Alafenamide Fumarate; COBI, Cobicistat; TDF, Tenofovir Disoproxil Fumarate; FTC, Emtricitabine; DRV, Darunavir; EVG, Elvitegravir; ART, antiretroviral therapy; INSTI, integrase strand transfer inhibitor; NNRTI, nonnucleoside reverse transcription inhibitor; NRTI, nucleoside Reverse Transcriptase Inhibitors; PI, protease inhibitor.

# **Table S2**: The list of gene set terms used in genome-wide association studies.

| **Category** | **Lipids** | **Body composition** | **Kidney function** |
| --- | --- | --- | --- |
| **Search terms** | ***ldl*** | ***waist*** | ***glomerular filtration rate*** |
|  |  | ***body fat*** |  |
|  |  | ***trunk fat mass*** |  |
| **GWAS terms** | LDL cholesterol levels in drinkers | Waist circumference adjusted for BMI | Glomerular filtration rate in chronic kidney disease |
|  | LDL cholesterol levels | Waist circumference variance | Estimated glomerular filtration rate in non-diabetics |
|  | LDL peak particle diameter | Waist circumference adjusted for BMI in non-smokers | Estimated glomerular filtration rate |
|  | LDL cholesterol levels in HIV | Waist circumference adjusted for BMI (adjusted for smoking) | Glomerular filtration rate in non-diabetics |
|  | LDL cholesterol | Waist-to-hip ratio adjusted for BMI | Estimated glomerular filtration rate in diabetes |
|  |  | Body fat mass | Glomerular filtration rate |
|  |  | Waist circumference adjusted for BMI (joint analysis main effects and physical activity interaction) |  |
|  |  | Waist circumference adjusted for BMI in active individuals |  |
|  |  | Body fat percentage |  |

# **Table S3**: Enrichment of the host-HIV interaction genes among ART transcriptional signatures across the cell lines.

| **Drug** | **P-value** | | |  |
| --- | --- | --- | --- | --- |
|  | **NES** | **Unadjusted** | **BF-adjusted** | **Group** |
| Abacavir | 5.173473384 | 2.30E-07 | 3.90629E-06 | a |
| Bictegravir | 4.904762227 | 9.35E-07 | 1.59019E-05 | a |
| Cobicistat | 0.241620141 | 0.809074514 | 1 | a |
| Darunavir | 6.008958559 | 1.87E-09 | 3.17422E-08 | a |
| Dolutegravir | 3.108571236 | 0.001879943 | 0.031959034 | a |
| Efavirenz | 5.315197347 | 1.07E-07 | 1.81121E-06 | a |
| Elvitegravir | 1.58531425 | 0.112894953 | 1 | a |
| Emtricitabine | 0.788528459 | 0.430387659 | 1 | a |
| Ritonavir | 2.584278335 | 0.009758301 | 0.165891115 | a |
| Tenofovir Alafenamide Fumarate | 5.939984869 | 2.85E-09 | 4.84582E-08 | a |
| Tenofovir Disoproxil Fumarate | 2.29653077 | 0.021645552 | 0.367974382 | a |
| Abacavir\|Dolutegravir\|Emtricitabine | 0.816517291 | 0.414204338 | 1 | b |
| Bictegravir\|Emtricitabine\|Tenofovir Alafenamide Fumarate | 4.968900543 | 6.73E-07 | 1.14467E-05 | b |
| Cobicistat\|Elvitegravir\|Emtricitabine\|Tenofovir Alafenamide Fumarate | 5.788737838 | 7.09E-09 | 1.20559E-07 | b |
| Darunavir\|Emtricitabine\|Ritonavir\|Tenofovir Disoproxil Fumarate | 2.234648641 | 0.025440425 | 0.432487223 | b |
| Dolutegravir\|Emtricitabine\|Tenofovir Alafenamide Fumarate | 5.738943423 | 9.53E-09 | 1.61957E-07 | b |
| Efavirenz\|Emtricitabine\|Tenofovir Disoproxil Fumarate | 4.174077042 | 2.99E-05 | 0.000508634 | b |

Enrichment of HIV related genes in gene activity signatures using blitzGSEA with BF, Bonferroni-corrected P-values. t-test is computed on NES values of single drugs (group a) vs combination drugs (group b).

|  | up regulated | < 0.05 BF |
| --- | --- | --- |
|  | down regulated | < 0.05 BF |

# **Table S4**: GWAS enrichment for lipid profile, body weight-related traits, and kidney phenotype by ART regimen in different cell types.

| **ART regimen** | **Drug composition** | **ART Acronym** | **LDL**  **HEPG2** | **LDL**  **THP-1** | **Body composition HEPG2** | **eGFR**  **HK2** |
| --- | --- | --- | --- | --- | --- | --- |
| Abacavir | Single drug | ABC | 0.149 | 0.667 | **0.007** | 0.872 |
| Bictegravir | Single drug | BIC | **-** | 0.673 | **-** | **0.000** |
| Cobicistat | Single drug | COBI | **0.010** | 0.612 | 0.880 | **0.000** |
| Darunavir | Single drug | DRV | 0.343 | 0.612 | 0.646 | **0.006** |
| Dolutegravir | Single drug | DTG | 0.249 | 0.619 | 0.558 | **0.011** |
| Efavirenz | Single drug | EFV | **0.002** | **0.017** | 0.635 | 0.148 |
| Elvitegravir | Single drug | EVG | **-** | **0.009** | **-** | - |
| Emtricitabine | Single drug | FTC | **0.002** | 0.562 | **0.028** | **0.003** |
| Ritonavir | Single drug | RTV | **0.000** | 0.561 | 0.777 | **0.000** |
| Tenofovir Alafenamide Fumarate | Single drug | TAF | 0.112 | 0.561 | **0.033** | **0.000** |
| Tenofovir Disoproxil Fumarate | Single drug | TDF | **0.023** | 0.777 | **0.041** | 0.872 |
| Abacavir\|Dolutegravir\|Emtricitabine | Combination drug | ABC\|DTG\|FTC | **0.021** | 0.849 | **0.033** | 0.398 |
| Bictegravir\|Emtricitabine\|Tenofovir Alafenamide Fumarate | Combination drug | BIC\|FTC\|TAF | 0.488 | 0.667 | 0.069 | **0.000** |
| Cobicistat\|Elvitegravir\|Emtricitabine\|Tenofovir Alafenamide Fumarate | Combination drug | COBI\|EVG\|FTC\|TAF | 0.083 | 0.667 | **0.040** | **0.000** |
| Darunavir\|Emtricitabine\|Ritonavir\|Tenofovir Disoproxil Fumarate | Combination drug | DRV\|FTC\|RTV\|TDF | **0.002** | 0.673 | **0.041** | 0.872 |
| Dolutegravir\|Emtricitabine\|Tenofovir Alafenamide Fumarate | Combination drug | DTG\|FTC\|TAF | - | 0.667 | - | **0.000** |
| Efavirenz\|Emtricitabine\|Tenofovir Disoproxil Fumarate | Combination drug | EFV\|FTC\|TDF | **0.008** | 0.667 | **0.028** | 0.872 |

LDL, low density lipoprotein, eGFR, estimated glomerular filtration rate. False discovery rate (FDR)-adjusted P-values are shown.

# **References**

1. Reagan-Shaw S, Nihal M, Ahmad N. Dose translation from animal to human studies revisited. FASEB J 2008;22:659–61.

2. Iulini M, Maddalon A, Galbiati V, et al. In vitro identification of drugs inducing systemic hypersensitivity reactions known in vivo to be associated with specific HLA genotypes. Toxicol In Vitro 2020;68:104953.

3. Bade AN, McMillan JM, Liu Y, et al. Dolutegravir Inhibition of Matrix Metalloproteinases Affects Mouse Neurodevelopment. Mol Neurobiol 2021;58:5703–5721.

4. Walsh JG, Reinke SN, Mamik MK, et al. Rapid inflammasome activation in microglia contributes to brain disease in HIV/AIDS. Retrovirology 2014;11:35.

5. James AM, King JR, Ofotokun I, et al. Uptake of tenofovir and emtricitabine into non-monocytic female genital tract cells with and without hormonal contraceptives. J Exp Pharmacol 2013;5:55–64.

6. Foster EG, Palermo NY, Liu Y, et al. Inhibition of matrix metalloproteinases by HIV-1 integrase strand transfer inhibitors. Front Toxicol 2023;5:1113032.

7. Pou J, Rebollo A, Roglans N, et al. Ritonavir increases CD36, ABCA1 and CYP27 expression in THP-1 macrophages. Exp Biol Med (Maywood) 2008;233:1572–82.

8. Porcal W, Hernandez P, Boiani M, et al. In vivo anti-Chagas vinylthio-, vinylsulfinyl-, and vinylsulfonylbenzofuroxan derivatives. J Med Chem 2007;50:6004–15.

9. Koyama T, Sun B, Tokunaga K, et al. DNA damage enhances integration of HIV-1 into macrophages by overcoming integrase inhibition. Retrovirology 2013;10:21.

10. de Baar MP, de Rooij ER, Smolders KG, et al. Effects of apricitabine and other nucleoside reverse transcriptase inhibitors on replication of mitochondrial DNA in HepG2 cells. Antiviral Res 2007;76:68–74.

11. Saida SJ, Manikandan A, Kaliyaperumal M, et al. Identification, isolation and characterization of dolutegravir forced degradation products and their cytotoxicity potential. J Pharm Biomed Anal 2019;174:588–594.

12. Hariparsad N, Nallani SC, Sane RS, et al. Induction of CYP3A4 by efavirenz in primary human hepatocytes: comparison with rifampin and phenobarbital. J Clin Pharmacol 2004;44:1273–81.

13. Moure R, Domingo P, Villarroya J, et al. Reciprocal Effects of Antiretroviral Drugs Used To Treat HIV Infection on the Fibroblast Growth Factor 21/beta-Klotho System. Antimicrob Agents Chemother 2018;62.

14. Kirkwood-Johnson L, Katayama N, Marikawa Y. Dolutegravir Impairs Stem Cell-Based 3D Morphogenesis Models in a Manner Dependent on Dose and Timing of Exposure: An Implication for Its Developmental Toxicity. Toxicol Sci 2021;184:191–203.

15. Xu C, Desta Z. In vitro analysis and quantitative prediction of efavirenz inhibition of eight cytochrome P450 (CYP) enzymes: major effects on CYPs 2B6, 2C8, 2C9 and 2C19. Drug Metab Pharmacokinet 2013;28:362–71.

16. Murphy RA, Stafford RM, Petrasovits BA, et al. Establishment of HK-2 Cells as a Relevant Model to Study Tenofovir-Induced Cytotoxicity. Int J Mol Sci 2017;18.

17. Blasi M, Balakumaran B, Chen P, et al. Renal epithelial cells produce and spread HIV-1 via T-cell contact. AIDS 2014;28:2345–53.

18. Iskandarmudasyah A, Louisa M, Arleni, et al. Influence of primaquine and ritonavir interaction on CYP3A4 mRNA expression in HepG2 cell culture. Medical Journal of Indonesia 2012;21:3–7.
